# The hypothalamus to brainstem circuit suppresses late-onset body weight gain

**DOI:** 10.1038/s41598-019-54870-z

**Published:** 2019-12-04

**Authors:** Yuko Maejima, Shigeki Kato, Shoichiro Horita, Yoichi Ueta, Seiichi Takenoshita, Kazuto Kobayashi, Kenju Shimomura

**Affiliations:** 1 Department of Bioregulation and Pharmacological Medicine, Fukushima Medical University School of Medicine, Fukushima, 960-1295 Japan; 2 Department of Molecular Genetics, Institute of Biomedical Science, Fukushima Medical University School of Medicine, Fukushima, 960-1295 Japan; 3 Department of Physiology, School of Medicine, University of Occupational and Environmental Health, Kitakyushu, 807-8555 Japan; 4 Advanced Clinical Research Center, Fukushima Global Medical Science Center, Fukushima Medical University, Fukushima, 960-1295 Japan

**Keywords:** Neural circuits, Ageing

## Abstract

Body weight (BW) is regulated in age-dependent manner; it continues to increase during growth period, and reaches a plateau once reaching adulthood. However, its underlying mechanism remains unknown. Regarding such mechanisms in the brain, we here report that neural circuits from the hypothalamus (paraventricular nucleus: PVN) to the brainstem (dorsal vagal complex: DVC) suppress late-onset BW gain without affecting food intake. The genetic suppression of the PVN-DVC circuit induced BW increase only in aged rats, indicating that this circuit contributes to suppress the BW at a fixed level after reaching adulthood. PVN neurons in the hypothalamus were inactive in younger rats but active in aged rats. The density of neuropeptide Y (NPY) terminal/fiber is reduced in the aged rat PVN area. The differences in neuronal activity, including oxytocin neurons in the PVN, were affected by the application of NPY or its receptor inhibitor, indicating that NPY is a possible regulator of this pathway. Our data provide new insights into understanding age-dependent BW regulation.

## Introduction

Body weight (BW) is regulated in an age-dependent manner. During the growth period, BW continues to increase as stature increases. Once adulthood is reached, growth is terminated and BW is typically set at approximately the same level throughout the remainder of one’s life^1,2^.

However, it remains unclear as to how BW is regulated at the most suitable level for its age. The main factor that regulates growth is growth hormone (GH). Secreted from the anterior pituitary, GH stimulates the production of insulin-like growth factor 1 (IGF-1) in the liver, and promotes chondrogenesis in the growth plate of the bone, which in turn induces longitudinal bone growth^3–5^. Upon reaching adulthood, GH and IGF-1 eventually decline, and stature growth reaches a plateau, shifting from the growth phase to the maintenance phase.

Generally, BW increase is associated only with stature growth. However, recent studies have reported age-dependent changes of neuronal properties in the areas of the brain that regulate food intake and energy expenditure^6–9^. Therefore, the existence of a brain circuit that regulates BW from the growth phase to the maintenance phase is possible. The expected brain neural circuit for BW maintenance would be to receive/integrate peripheral metabolic information, which would be output as whole body regulation^10–12^.

The paraventricular nucleus (PVN) is an essential component for integrating energy homeostasis^10,13^, and is composed of numerous kinds of neurons, such as oxytocin (Oxt), corticotrophin releasing hormone (CRH), arginine vasopressin (AVP), and NUCB2/Nesfatin-1 neurons^10,14^. Oxt, AVP, and CRH neurons project to the caudal brainstem directly^15–17^, and function as “anorexigenic factors” or “negative energy balance” factors^15,18–20^. The PVN receives strong projections from the arcuate nucleus (ARC), the neurons of which are known as first order neurons that sense circulating peripheral signals such as insulin, leptin and ghrelin^18^. Neuropeptide Y (NPY) and α-melanocyte stimulating hormone (α-MSH), derived from the precursor proopiomelanocortin (POMC), are major neuronal peptides for regulating appetite in the ARC as orexigenic and anorexigenic peptides, respectively. Hence, these two neuronal species in the ARC provide inhibitory or stimulatory signals to the PVN neurons, thereby integrating energy state information from peripheral signals.

We previously reported on a projection from the PVN in the hypothalamus to the nucleus of the solitary tract (NTS), which is a component of the dorsal vagal complex (DVC) in the brainstem that regulates energy homeostasis, including food intake^14,15,21^. The PVN receives/integrates peripheral metabolic information from neurons in the ARC^18,22^ and outputs to the brainstem nuclei, including the DVC, which regulates the gastrointestinal organs via vagal efferent output for food intake and BW gain^23^. Therefore, the PVN-DVC circuit is a candidate circuit that may regulate BW in an age-dependent manner.

In the present study, we used a genetically-induced tetanus neurotoxin to block the PVN-DVC circuit using a double-infection technique^24^. We tested whether this circuit functions as a regulator of BW gain, and revealed that blocking the PVN-DVC circuit induces continuous BW increase even after termination of the growth phase. Additionally, this effect was independent from the amount of food intake and stature growth. Furthermore, electrophysiological analysis of neurons in the PVN, where the somata of the PVN-DVC circuit reside, revealed that these neurons become more active after reaching the maintenance phase, indicating that activation of this circuit after reaching adulthood may terminate BW increase.

These data have implications for understanding both the mechanism of growth regulation, as well as a possible etiology of obesity development.

## Results

### Long-term blockage of the PVN-DVC circuit results in continuous BW increase

**Figure 1:**
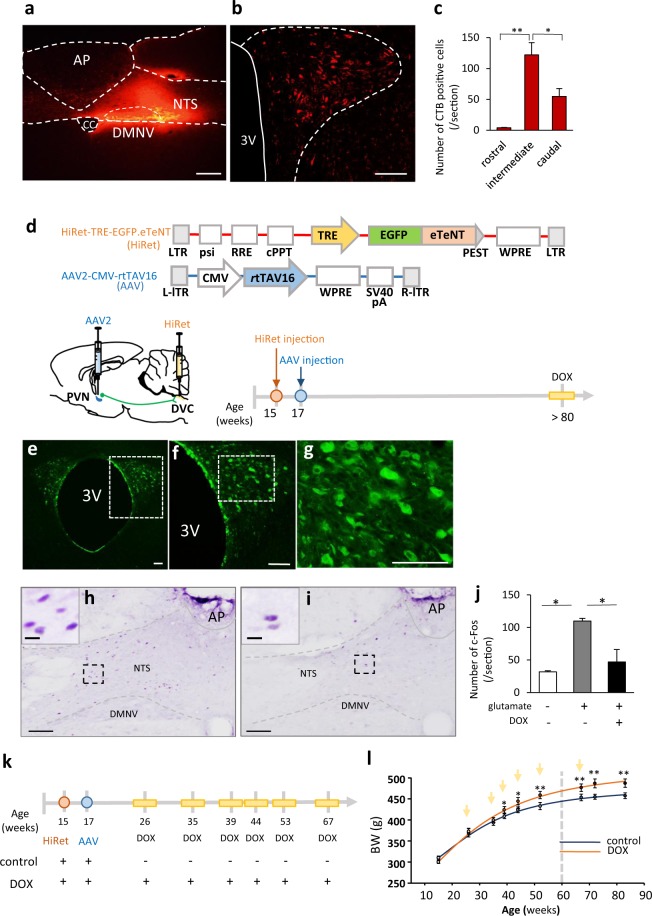
The physiological role of the PVN-DVC circuit. (**a**) The CTB-injected area in the DVC. AP: area postrema, NTS: nucleus of the solitary tract, DMNV: dorsal motor nucleus of the vagus, CC: central canal. (**b**) The distribution of CTB-positive neurons in the PVN. 3 V: third ventricle. (**c**) The number of CTB-positive neurons per section in each region of the PVN. *P < 0.05, **P < 0.01, one-way ANOVA followed by Tukey’s multiple range test. *n* = 4. Error bars indicate s.e.m. Scale bars in (**a**) and (**b**) indicate 100 μm. (**d**) The construction of viral vectors (upper panel), scheme of viral injection site, and experimental procedure for confirmation of virus expression in PVN neurons (lower panel). AAV were injected into the intermediate region of the PVN due to the abundance of CTB-positive neurons. At the end of the experiment, after two days of continuous intraperitoneal DOX injection, the animals (>80 weeks old) were perfused to confirm EGFP expression. (**e**) Distribution of EGFP-expression in the PVN. (**f**) Enlarged image of the dotted area in (**e**). Scale bar indicates 100 μm. (**g**) Enlarged image of the dotted area in Figure f. Scale bar indicates 100 μm. (**h–j**) The functional examination with suppression of the PVN-DVC circuit. (**h**) Representative image of c-Fos expression in the DVC after intra-PVN glutamate injection without DOX treatment. (**i**) Representative image of c-Fos expression in the DVC after intra-PVN injection of glutamate in DOX-injected rats. Scale bar indicates 100 μm. The images in the left corner of h and i indicate enlarged images of the dotted areas in each image. Scale = 10 μm (**j**) The number of c-fos positive neurons per section in the NTS of each treatment. *P < 0.05, one-way ANOVA followed by Tukey’s multiple range test. (*n* = 4, 3, 4). (**k–i**) Scheme of the experimental procedure including virus injection and DOX administration (k) and BW change followed by DOX administration (**l**). The curve was best-fit to the following equation: Y = 523.5–285.2/(1 + [x/28.7]^2^) for the control group and Y = 476.5-204.2/(1 + [x/28.7]^2.3^) for the DOX-injected group. *n* = 5, 6. Arrows indicate the timing of DOX or saline injection. The dotted line indicates age 60 weeks. *P < 0.05, **P < 0.01. Two-way ANOVA followed by Tukey’s multiple range test (F_1, 72_ = 57.55, P < 0.01).

We first confirmed the presence of the PVN-DVC circuit in rats by injecting cholera toxin B into the DVC area (Fig. 1a,b). The injection sites for each rat are shown in Supplementary Figure S1. Anatomically, the rostral, intermediate and caudal parts of the PVN were defined as −0.92 mm to −1.60 mm, −1.61 mm to −1.88 mm, and −1.89 to −2.12 mm from bregma, respectively. This definition was decided based on the morphological character of the PVN and positional relationship to another nuclei. In addition, 67.7 ± 3.2% (*n* = 4) of neurons that project to the DVC were distributed in the intermediate part of the PVN (Fig. 1c). We then genetically suppressed this circuit specifically by blocking synaptic transmission. A highly efficient retrograde gene transfer (HiRet) lentiviral vector, carrying an enhanced tetanus neurotoxin light chain (eTeNT) and enhanced GFP (HiRet-TRE-EGFP.eTeNT)^24^, was injected into the bilateral DVC (Fig. 1d). Similarly, an adeno-associated virus serotype 2 (AAV2) vector carrying the Tet-on sequence, a variant of the reverse tetracycline transactivator (rtTAV16) (Fig. 1d) that is generated under the control of the cytomegalovirus (CMV) promotor (AAV2-CMV-rtTAV16), was injected bilaterally into the intermediate regions of the PVN. With this technique, induced eTeNT depresses synaptic transmission in the PVN-DVC circuit neurons during the external administration of doxycycline (DOX)^24^. EGFP expression was detected in the PVN neurons (Fig. 1e–g). Functionally, intra-PVN injection of glutamate induced c-Fos expression in the NTS of the control group without DOX treatment (109.9 ± 3.9/section, *n* = 3) (Fig. 1h,j) whereas it reduced c-Fos expression in the NTS of the DOX-treated circuit depressed group (46.8 ± 19.0/section, *n* = 4) (Fig. 1i,j). This was the same level of c-Fos expression as that observed in the intra-PVN saline injected group (31.7 ± 1.7/section, *n = *4) (Fig. 1j). Therefore, in the present study, infected neurons in the PVN functioned to suppress synaptic transmission from the PVN to the DVC under DOX treatment.

PVN-DVC circuit depression by intraperitoneal injection of DOX was performed as shown in Fig. 1k. By plotting the whole BW change on a continuous scale, we found that BW in control rats rapidly increased in the first 30 weeks, before gradually reaching a plateau at around 60 weeks (Fig. 1l) (control: saline intraperitoneal (i.p.) injected group without PVN-DVC circuit depression). However, the DOX treated PVN-DVC circuit-depressed rats showed continuous BW increase even after 60 weeks (Fig. 1l), irrespective of body length (Supplementary Figs. S2a–c), indicating that this circuit only regulates BW in a fixed state in aged (>60 weeks) rats. Significant differences in BW between the control and DOX groups were observed after 40 weeks. In order to examine the effect of blocking the PVN-DVC circuit on body composition, rats aged 47–59 weeks old were injected with HiRet-TRE-EGFP.eTeNT into the NTS. After two weeks, the PVN of these rats was then injected with AAV2-CMV-rtTAV16. When we compared body fat compositions, obtained by CT scanning before and after DOX treatment in the control and DOX groups aged >50 weeks, the repetitive DOX-treated circuit suppressed group showed significant increases in visceral fat and body fat percentage, as well as slight but significant increases in muscle weight, compared with the pre-DOX treatment state (Supplementary Fig. S3).

### Age-dependent effects of PVN-DVC block and electrophysiological properties of PVN neurons

**Figure 2:**
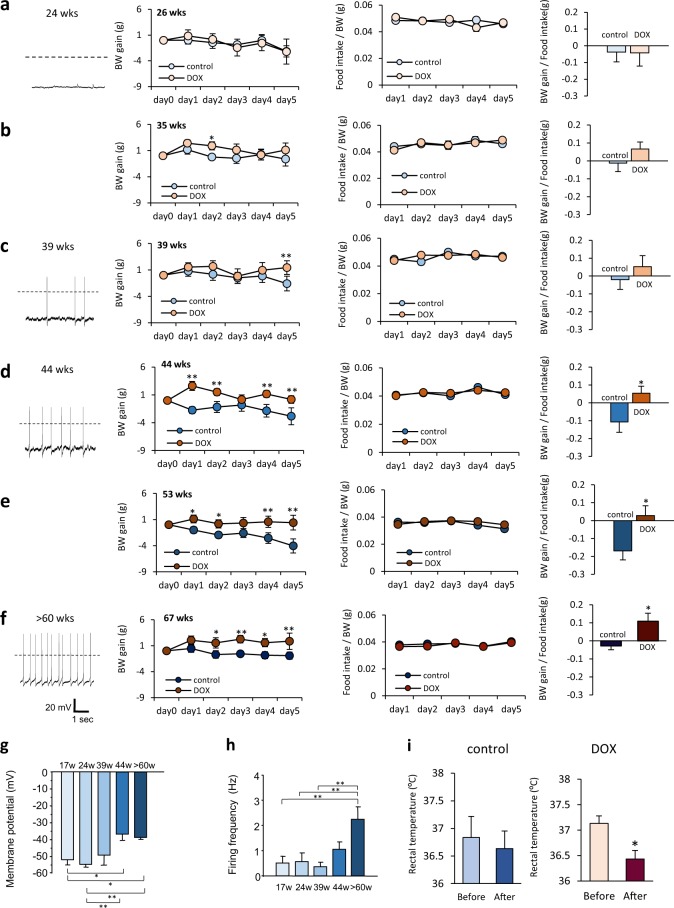
Age-dependent effects of the reversible blockade of the synaptic transmission from the PVN to the DVC. (**a–f**) left: Representative recordings of the electrical activity of PVN neurons in the brain slices at ages 24 (*n* = 17) (**a**), 39 (*n* = 10) (**c**), 44 (*n* = 10) (**d**), and >60 (*n* = 13) weeks (**f**). Middle left and middle right: BW gain (middle left panel), food intake/BW (middle right panel) during intraperitoneal DOX injection at 26 weeks (BW gain, [F_1, 70_ = 0.117, P > 0.05]; food intake, [F_1, 56_ = 0.003, P > 0.05]) (*n* = 8, 8) (**a**), 35 weeks (BW gain, [F_1, 70_ = 7.633, P < 0.01]; food intake, [F_1, 56_ = 0.009, P > 0.05]) (*n* = 7, 9) (**b**), 39 weeks (BW gain, [F_1, 75_ = 7.779, P < 0.05]; food intake,[F_1, 60_ = 0.226, P > 0.05]) (*n* = 8, 9) (**c**), 44 weeks (BW gain, [F_1, 65_ = 41.042, P < 0.01]; food intake, [F_1, 52_ = 0.058, P > 0.05]) (*n* = 7, 8) (**d**), 53 weeks (BW gain, [F_1, 75_ = 31.798, P < 0.01]; food intake, [F_1, 60_ = 2.132, P > 0.05]) (*n* = 8, 9) (**e**), and 67 weeks old (BW gain, [F_1, 30_ = 25.738, P < 0.01]; food intake, [F_1, 30_ = 1.610, P > 0.05]) (*n* = 4, 4) (f). *P < 0.05, **P < 0.01. Two-way ANOVA followed by Tukey’s multiple range test. right panel: Food efficacy (average calculated from BW gain/food intake during DOX administration) at 26 (**a**), 35 (**b**), 39 (**c**), 44 (**d**), 53 (**e**), and 67 (**f**) weeks old. Unpaired *t*-test. (**g,h**) The membrane potential in the PVN neurons at 17, 24, 39, 44, and >60 weeks old. *P < 0.05, **P < 0.01, one-way ANOVA followed by Tukey’s multiple range test. *n* = 9–15 (**g**). The firing frequency in the PVN neurons at 17, 24, 39, 44, and >60 weeks old. **P < 0.01, one-way ANOVA followed by Tukey’s multiple range test. *n* = 9–15 (**h**). (**i**) Rectal temperature before (Day 0) and after (Day 3) the saline (left panel, control) or DOX (right panel) injection. *P < 0.05, paired t-test. *n* = 3 each.

During each DOX-injected period, the 26-week-old rats showed no significant differences in BW change, food intake, or food efficacy (FE: BW gain/Food intake), compared to the control group (Fig. 2a, middle-right). DOX injection at ages 35 and 39 weeks also yielded almost no effect on BW change, food intake, or FE (Fig. 2b,c middle-right). However, when the PVN-DVC neural circuit was depressed in rats aged ≥44 weeks, an obvious increase in BW was observed without affecting food intake on a daily basis (Fig. 2d–f middle-right). In addition, food intake was not affected on an hourly basis when measured after overnight (14 h) fasting (Supplementary Fig. S4a,b).

Interestingly, neurons in the PVN, where the neuronal origin of this circuit resides, showed a different electrical activity depending on age. The PVN neurons of the younger rats (17 weeks and 24 weeks) were hyperpolarized (with an average membrane potential of approximately −50 mV) with very little action potential firing (Fig. 2a left, g,h), whereas the PVN neurons of the aged rats (44 weeks and >60 weeks) showed significantly more depolarized membrane potentials (mean −37 mV for 44 weeks, −38 mV for >60 weeks) with spontaneous firing (1.1 Hz for 44 weeks and 2.2 Hz for >60 weeks) (Fig. 2d,f left, g,h). These results indicate that an age of around 40 weeks, corresponding to approximately 25 years of age in humans^25^, is the approximate point at which the PVN-DVC circuit shifts its activatory properties in rats.

Increases in membrane potential and firing frequency in PVN neurons were observed even in younger rats when they were fed with a HFD for 24 hours (Supplementary Fig. S5) or for 8 weeks (Supplementary Fig. S6).

In the aged rats (>60 weeks), rectal temperature was reduced after PVN-DVC circuit suppression was induced by DOX treatment. However, no changes in rectal temperature were observed in the control group (Fig. 2i).

### NPY as an upstream regulator of age-dependent PVN neuron activity

**Figure 3:**
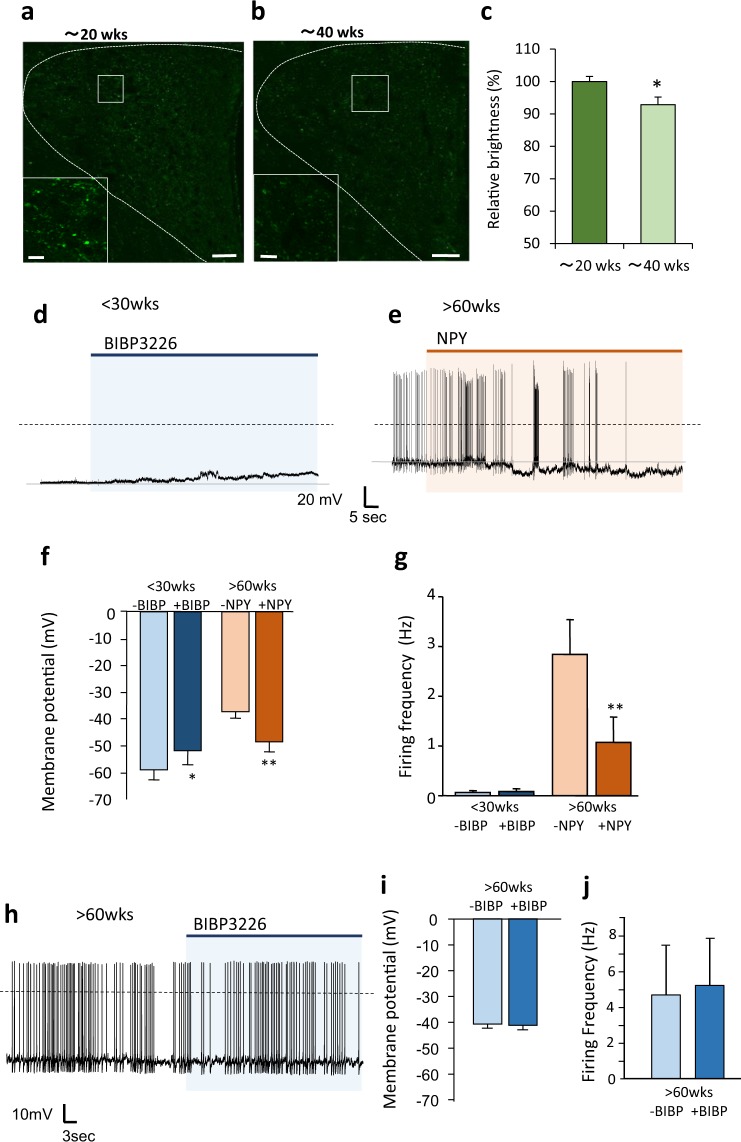
Age-dependent activation of PVN neurons regulated by NPY in the PVN. (**a–c**), Confocal image of distribution of NPY-immunoreactive terminals/fibers in the PVN of rats aged around 20 weeks (18–23 weeks old) (**a**) and 40 weeks (39–44). Scale bars indicate 50 μm. (**b**) The bottom left square images are the enlarged images of the white square in each image. Scale bars in the enlarged images indicate 10 μm. Relative brightness acquired from image analysis of the confocal images (**c**). The brightness of NPY immunofluorescence in the PVN of rats aged around 20 weeks old was generalized as 100% (*n* = 8 each). *P < 0.05. unpaired *t*-test. (**d**) The representative recording of electrical activity of PVN neurons from rats aged <30 weeks after NPY antagonist (BIBP3226; 10^−6^ M) application. (**e**) Representative recording of electrical activity of PVN neurons from rats aged >60 weeks after NPY (10^−8^ M) application. (**f**) The membrane potential in the PVN neurons before and after application of BIBP3226 or NPY in rats aged <30 weeks and those aged >60 weeks, respectively (*n* = 6 each). *P < 0.05, **P < 0.01. paired *t*-test. (**g**) The firing frequency in the PVN neurons before and after application of BIBP3226 and NPY in the rats aged <30 weeks and those aged >60 weeks, respectively (*n* = 6 each). **P < 0.01. paired *t*-test. (**h**) The representative recording of electrical activity of the PVN neurons from the rats aged >60 weeks after NPY antagonist (BIBP3226; 10^−6^ M) application. (**i–j**) The membrane potential (**i**) and firing frequency (**j**) in the PVN neurons before and after application of BIBP3226 in the rats aged >60 weeks.

We investigated a possible upstream factor that may be responsible for the age-dependent activation of PVN neurons. PVN neurons from the younger rats (<40 weeks) were inactive; therefore, we tested the influence of neuropeptide Y (NPY) and α-melanocyte-stimulating hormone (α-MSH), major neuropeptides, to show inhibitory and excitatory inputs on the PVN neurons, respectively^26–28^. First, we compared the NPY and α-MSH-immunoreactive (IR) terminals/fibers in the PVN of rats aged approximately 20 and 40 weeks old. We found a significant reduction of NPY IR terminal/fiber density of approximately 10% in the PVN of the 40-week-old rats compared to the 20-week-old rats (Fig. 3a–c), whereas α-MSH IR terminals/fibers in the PVN tended to decrease but not significantly different in the 40-week-old rats compared to the 20-week-old rats (Supplementary Figs. S7a–c). Thus, age-dependent activation of PVN neurons is unlikely to depend on the increment of excitatory input from α-MSH. Therefore, we focused on NPY, and tested the functional relevance of this age-related difference in NPY terminal/fiber density by measuring the impact of NPY on PVN neurons electrophysiologically. One of the most common NPY receptor subtypes in PVN neurons is the Y1 receptor^29^. When we applied the NPY Y1 receptor antagonist BIBP3226^30^ to PVN neurons of young rats (<30 w), the membrane potential was elevated but did not induce action potential firing (Fig. 3d,f and g). On the other hand, applying NPY to the activated PVN neurons of the aged rats (>60 w) hyperpolarized the membrane and reduced action potential firing in most of the measured neurons (83.3%) (Fig. 3e–g); however, the application of BIBP3226 had no effect on either membrane potential or action potential firing (Fig. 3h–j). These results indicate that PVN neuronal activity is different in young and aged rats in accordance with the inhibitory input induced by NPY. While the impact of NPY on PVN neuronal activity was evident in the aged rats, the activity in the young rats may have been strongly suppressed by multiple factors besides NPY, since despite BIBP3226 application alone increased membrane potential, it did not induce action potential firing. Additionally, because BIBP3226 showed no effect on the PVN neuronal activity of the aged rats, the impact of endogenous NPY should be less when compared to young rats.

### Oxytocin neurons as one of the components of the PVN-DVC circuit

**Figure 4:**
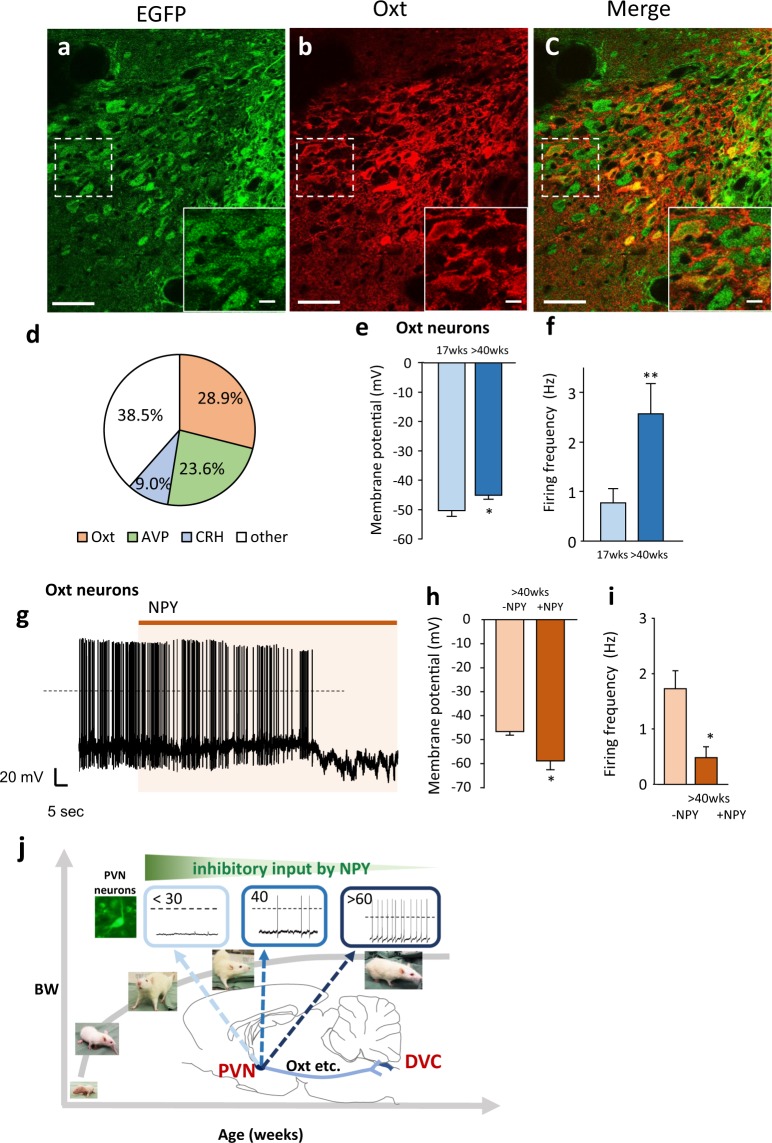
Oxytocin neurons as one of the components of the PVN-DVC circuit. (**a–c**) The distribution of the EGFP-expressing neurons (**a**), Oxt-positive neurons (**b**) and merged image of a and b (**c**) in the PVN after DOX treatment continuously for two days. Scale bars indicate 50 μm. The image located in the bottom right is an enlarged image of the dotted square in each image, in which the scale bars indicate 10 μm. 3 V = third ventricle. (**d**) Percentage of Oxt, AVP, and CRH neurons among EGFP expressing neurons. (*n* = 3–4). (**e**) The membrane potential in PVN Oxt neurons at 17 weeks and >40 weeks. *P < 0.05. unpaired *t*-test. *n* = 29, 19, each. (**f**) The firing frequency in PVN Oxt neurons at 17 weeks and >40 weeks. **P < 0.01. unpaired *t*-test. *n* = 29, 19, each. (**g**), The representative recordings of the electrical activity of PVN Oxt neurons under NPY (10^−8^ M) treatment in the brain slices at >40 weeks. (**h**) The membrane potential in the PVN Oxt neurons before and after the application of NPY in rats aged >40 weeks. *n* = 6. *P < 0.05. paired *t*-test. (**i**) The firing frequency in the PVN Oxt neurons before and after application of NPY in rats aged >40 weeks. *n* = 6. *P < 0.05. paired *t*-test. (**j**) Summary of the mechanism to regulate age-dependent BW. BW increase continues until around 60 weeks. PVN neurons are relatively inactive in young rats (<30 weeks). Action potential firing and membrane potential increases in adulthood (around 40 weeks old) due to decreasing inhibitory inputs from NPY neurons with age. Upstream and downstream factors that regulate age-dependent activity of the PVN include NPY and Oxt, respectively. The PVN-DVC circuit is considered a regulator of age-dependent BW under NPY influence.

In order to identify the neuronal species that compose the PVN-DVC circuit, three major peptides located in the PVN (Oxt, AVP, and CRH) were examined. As shown in Fig. 4a–c and Supplementary Figure S8a–f, Oxt, CRH, and AVP were co-localized with circuit composing DOX-induced EGFP-positive neurons (Fig. 4d). Inevitable injury that caused by the virus injection procedure, may have prevented effective staining even under colchicine treatment. But we found that approximately 30% of the EGFP-positive neurons were found to be Oxt neurons (Fig. 4d). We therefore analyzed Oxt neurons as one of the components of this neural circuit.

By using a transgenic rat model with targeted expression of monometric red fluorescent protein 1 in Oxt neurons (Oxt-mRFP rat), we identified the Oxt neurons in rat PVN brain slices and compared their electrophysiological properties in rats aged 17 weeks and >40 weeks. As shown in Fig. 4e–i, similar results were found regarding PVN-Oxt neurons: the >40-week-old rats’ Oxt neurons were more depolarized with spontaneous firing (Fig. 4e,f) and were able to become hyperpolarized with reduced action potential firing upon application of NPY (Fig. 4g–i).

## Discussion

In the current study, we reported data indicating the existence of a neural circuit for regulating late-onset BW gain, which contributes to maintaining BW depending on age (Fig. 4j). To date, the majority of research on BW regulation is focused on obesity, because of its global epidemic spread^31^. However, BW increase also occurs during the growth process as well as during obesity development. In the present study, we studied the contribution of the PVN-DVC circuit and aging to BW control by genetically suppressing this circuit. We injected AAV2-CMV-rtTAV16 into the PVN, as well as HiRet-TRE-EGFP.eTeNT into the DVC, and specifically blocked synaptic transmission of this circuit by i.p. injection of DOX. In the current study, we used same viral vectors injected rats with saline i.p. injection as a control group. This is because we confirmed that injection of glutamate into the PVN increased c-Fos expression in the DVC without DOX treatment (saline i.p. injection), but did not increase c-Fos expression with DOX treatment (Fig. 1j).

The suppression of the PVN-DVC circuit increased BW in rats even after the growth stage (>60 weeks). We measured PVN neuronal activity in rats of various ages, from young to aged stage. Although the neurons whose electrical activities we measured may not have necessarily composed the PVN-DVC circuit, our data indicate that the overall activities of neurons in the PVN (including neurons that project to the DVC) were increased in rats after the growth stage (>60 weeks), the same timing as that when the depression of the PVN-DVC circuit showed increase of BW.

We also consider that NPY is a possible regulator of this circuit (Fig. 4j). Consistent with the present data, it has been reported that NPY expression in the ARC, a major source of NPY input to the PVN, as well as the expression of the NPY Y1 receptor in the PVN, both decrease with age^6,32^.

Regarding BW increase after suppression of the PVN-DVC circuit, CT scanning demonstrated that BW increase was the result of an increase in fat mass, especially visceral fat mass (Supplementary Fig. S3). Considering the decline of body temperature (Fig. 2i), it is possible that activation of the PVN-DVC circuit may compensate the decline of body temperature due to aging. However, core body temperature is determined by the balance between heat production and heat loss. Thus, rectal temperature by itself cannot be an indicator of thermogenesis. Further study is required to investigate these points. However, considering the decline of rectal temperature and increased food efficacy after PVN-DVC circuit suppression, we consider that activation of the PVN-DVC circuit may increase energy expenditure, thus preventing fat accumulation. Because previous studies have reported correlations between fat mass, aging, and sympathetic nerve activity^33,34^, the results of the present study may indicate that age-dependent activation of the PVN-DVC circuit may be triggered by fat accumulation. Activation of sympathetic nerve activity contributes to the induction of lipolysis through β-adrenergic receptors in adipocytes^35^. However, activation of NPY neurons in the ARC has been reported to inhibit sympathetic nervous system outflow and inhibit lipolysis^35^. Therefore, the PVN-DVC circuit may prevent fat accumulation by activating sympathetic nerve activity under the regulation of NPY, and function as a compensatory mechanism to regulate late-onset BW gain. However, further study is required to investigate these points.

The AAV injected bilaterally into the PVN in our experiment may have spread within the PVN area and randomly infected the PVN neurons. Neurons with various neuropeptides are distributed in the PVN, such as Oxt, AVP, CRH, NUCB2/nesfatin-1 and thyrotropin-releasing hormone (TRH)^36^. Since the AAV was infected randomly in the PVN, any one of these neuropeptides may be responsible for BW gain after DOX induced depression of synapse transmission. Although we could not fully identify the species of neurons that compose the PVN-DVC circuit due to technical limitations, we found Oxt neurons to be one of the neuronal species identified in the current study. Following the discovery of leptin and ghrelin in the 1990s, vigorous research on obesity has been conducted. Through these studies, the possible anorexigenic effect of projection from the PVN to the nucleus of the solitary tract (NTS), which is a component of the DVC, has been reported^15,16,21^. Although these reports seem promising for obesity research, recent studies have reported contradictory results for the anorexigenic effect of the PVN-DVC/NTS circuit. The specific ablation of PVN Oxt neurons, which project to the DVC, has been reported to fail to show any effect on food intake and BW in mice given a normal chow diet^37^. Furthermore, a study by Stachniak, *et al*. also revealed that the PVN-NTS circuit has no effect on short-term food intake^38^. The present study confirmed that blocking the PVN-DVC circuit has no effect on food intake on an hourly or daily basis (Supplementary Fig. S4 and Fig. 2). Our data clearly show that the effect of the PVN-DVC circuit on BW regulation becomes clear only after long-term observation. The main physiological role of the PVN-DVC circuit may not be feeding regulation. The PVN-DVC circuit may contribute to the suppression of late-onset BW gain by regulating food efficacy. However, microinjection of Oxt into the NTS or fourth ventricle induced decline of food intake^39,40^, and the knockdown of Oxt receptors in the NTS induced hyperphagia and BW gain^41^. Taken together with the results of previous studies, it is possible that there are two subtypes in Oxt receptor expressing NTS neurons. One subtype may be neurons related to the regulation of energy expenditure; these neurons may receive projection from PVN Oxt neurons. The other subtype may be neurons related to feeding regulation, which do not receive projection from PVN Oxt neurons, but may sense Oxt in the bloodstream. In order to clarify this hypothesis, further studies on the specific depression of PVN Oxt neurons to the DVC circuit in the long term are required.

From an evolutionary point of view, the hypothalamus originates from the neurosecretory brain center in the Urbilateria, which connects sensory cues from the surrounding environment to changes in body physiology^42^. Likewise, in vertebrates, the main function of the feeding center in the hypothalamus is to connect peripheral energy status to changes in energy homeostasis^43,44^. Therefore, it is possible that development of obesity is a failure of the hypothalamus to regulate BW.

In support of this idea, when we intervened in young rat metabolism by continuously feeding rats a HFD, we found that PVN Oxt neurons were depolarized and activated, similar to the PVN neurons of aged rats (Supplementary Fig. S6a–c). In order to clarify the relationship between this phenomena and BW regulation, we measured BW, energy intake and PVN Oxt neuronal activity. On the first day after initiating the HFD in young rats, BW gain was decreased, whereas energy intake was increased, compared with the control rats (Supplementary Fig. S5a,b). We found that at this point, approximately half of the PVN Oxt neurons (eight out of 15 Oxt neurons) in the young rats were activated (Supplementary Fig. S5c,d). This result is consistent with those of a previous study, which reported that mice with ablated PVN Oxt neurons showed a higher increase in BW compared to control mice, without affecting food intake, only when they were given a HFD^37^. This indicates that this circuit may also function to recover normal BW when the body’s metabolism is significantly disturbed. Our data indicate that sudden HFD application that disturbs the body’s metabolism may induce activation of PVN neurons to compensate and recover normal BW, which ultimately leads to the reduction of BW one day after starting a HFD. However, continuous HFD may overcome this compensatory mechanism, which also suggests the risk of continuous HFD consumption. Therefore, it is possible that the physiological relevance of the PVN-DVC circuit at a younger age is to regulate normal BW, as well as age-dependent BW increase.

Our electrophysiological data show that membrane potential and firing frequency both increase in PVN neurons in an age-dependent manner. In the younger rats, the NPY receptor 1 and 5 blocker increased only membrane potential, and did not induce action potential firing. This result suggests that there are other factors that suppress PVN neurons. On the other hand, in aged rats (>60 weeks), increased firing frequency and membrane potential in PVN neurons, including oxytocin neurons, decreased with NPY application, but no effect was observed with BIBP3226 application. These data indicate that the contributions of NPY and NPY-Y1R were more prominent in the PVN neurons of the young rats, compared with the aged rats. Because NPY-Y1 receptor is not expressed in all PVN neurons, and also GABAergic synaptic response was reported to be observed in neurons in the PVN^26^, NPY may inhibit presynaptic GABA release, and may activate PVN neurons. However, because most PVN neurons (83.3%) in the aged rats responded to NPY in the present study, our results suggest that PVN neuronal activation in aged rats should mainly be due to the reduction of inhibitory NPY input.

The current study has some limitations. First, there is a possibility that our double infection PVN-DVC circuit depression technique may have depressed additional projection from the PVN to different sites due to axon collateral projection. In addition, we previously demonstrated that single PVN neurons project at least to the DVC and ARC with axon collaterals^14^. The double infection technique used in the present study prevents synapse transmission from the synaptic terminals^24^. If the double-infected neurons had axon collaterals, all collateral synapse transmissions may have been suppressed. However, the percentage of axon collateral neurons within the PVN-DVC circuit is not clear. Therefore, further study is required to determine the contribution of axon collateral projection in the present study.

Collectively, our data show that the PVN-DVC circuit functions to suppress late-onset BW gain. The possible relevance of these results for humans is that this circuit may control normal BW, and its failure may lead to the development of obesity. This circuit may therefore be a target for obesity prevention, by restoring normal BW.

## Methods

### Animals

Male Wistar rats, purchased from Japan SLC, or monometric red fluorescent protein 1 in Oxt neurons (Oxt-mRFP rat)^45^, a gift from Prof. Ueta from the University of Occupational and Environmental Health, were housed on a 12-h light cycle (07:00–19:00). The animals were given conventional food (CE-2; Clea) and water *ad libitum*. All experimental procedures and care of animals were carried out according to the relevant guidelines and regulations, and were approved by the Fukushima Medical University Institute of Animal Care and Use Committee.

### Stereotaxic surgery and injection coordinates

HiRet-TRE-EGFP.eTeNT and AAV2-CMV-rtTAV16 were generated at Fukushima Medical University as previously described^24^. Rats aged 15 weeks were anesthetized and positioned on a stereotaxic alignment device (David Kopf). HiRet-TRE-EGFP.eTeNT was injected into the bilateral dorsal vagal complex (DVC; targeted 14.0 mm caudal to bregma, 0.6 mm lateral to the midline, and 7.8 mm below the skull surface with fine adjustment). The injection was performed using a modified glass pipette and syringe pump (injection volume and speed: 0.5 μl/5 min). After injection, the glass pipette was kept in the injection site for 10 min to prevent spreading of the HiRet-TRE-EGFP.eTeNT-containing solution to outside the DVC. Two weeks after the operation, AAV2-CMV-rtTAV16 was injected bilaterally into the PVN of the same rats (1.8 mm caudal to bregma, ±0.3 mm lateral from the midline, and 7.8 mm below the skull) (injection volume and speed: 0.5 μl/5 min). After injection, the glass pipette was kept in the injection site for 10 min to prevent spreading of the AAV2-CMV-rtTAV16-containing solution to the outside of the PVN. This double vector system interrupts synaptic transmissions from the PVN to the DVC, which is a reversible technique for blocking neural transmissions, and does not depend on cell-specific promoters or a transgenic technique^24^. The retrograde tracer cholera toxin subunit B Alexa Fluor 594 (CTB 594; 0.5 μl of 0.5 mg/ml) (Invitrogen) was used to identify the pathways connecting the DVC (targeted to 14.0 mm caudal to bregma, 0.6 mm lateral to the midline, and 7.8 mm below the skull surface with fine adjustment). The titers of HiRet-TRE-EGFP.eTeNT and AAV2-CMV-rtTAV16 were 1.26 × 10^12^ copies/ml and 8.79 × 10^13^ copies/ml, respectively.

We used rats injected with HiRet-TRE-EGFP.eTeNT into the DVC and AAV2-CMV-rtTAV16 into the PVN. The DOX-treated rats were considered an experimental group, and the saline-treated (0.9% NaCl) rats were considered the control group.

### Lateral ventricular and intra-PVN cannulation

For lateral ventricle injection, a 26-gauge guide cannula was placed stereotaxically into the lateral ventricle (0.8 mm caudal to bregma, 1.5 mm lateral, and 3.5 mm below the surface of the skull). For intra-PVN injection, the cannula was inserted into the PVN (1.8 mm caudal to bregma in the midline, 0.3 mm lateral, and 8.0 mm below the surface of the skull). The injector needle extended 0.1 mm beyond the tip of the guide cannula.

### Identification of CTB labeled neurons

Seven days after CTB injection, the rats were perfused with 4% paraformaldehyde (PFA) containing 0.2% picric acid. Then, 40-μm-thick brain sections were cut using a freezing microtome. Sections at 160 μm intervals between −1.3 and −2.0 mm from bregma were mounted on glass slides. Confocal fluorescence images of the CTB-labeled neurons were acquired with a confocal laser-scanning microscope (FV10i; Olympus). The accuracy of each injection was confirmed histologically, and the CTB-positive neurons were counted on the fluorescence images.

### Identification of EGFP labeled neurons

At the end of the experiment, after intraperitoneal (i.p.) injection of doxycycline (DOX) (SIGMA-ALDRICH; 10 mg/kg/10 ml) for two consecutive days, the HiRet-TRE-EGFP.eTeNT- and AAV2-CMV-rtTAV16-infected rats were perfused with 4% PFA containing 0.2% picric acid. Then, 40-μm-thick brain sections were cut using a freezing microtome. Sections at 160 μm intervals between −1.3 and −2.0 mm, and between −13.3 and −14.6 mm from bregma were used for EGFP immunostaining. In the brains of the aged rats, lipofuscin accumulated and autofluorescence appeared. In order to quench lipofuscin autofluorescence, the brain sections were pretreated with TrueBlack for 8 min (Biotium). After this treatment, the sections were incubated with blocking solution (2% bovine serum albumin, 5% normal goat serum) for 1 h. After blocking, the sections were incubated with rabbit anti-EGFP antibody (1:1000, A-6455, Thermofisher). The sections were then rinsed with PBS and incubated with Alexa Fluor 488-labeled goat anti-rabbit IgG (1:500: Life Technologies) for 40 min. Finally, fluorescence images were acquired using a fluorescence microscope (Olympus).

For double staining of EGFP and Oxt, AVP, CRH, the HiRet-TRE-EGFP.eTeNT and AAV2-CMV-rtTAV16, 33-week-old, double infected rats with lateral ventricle cannulation were intraperitoneally injected with doxycycline (DOX) (SIGMA-ALDRICH; 10 mg/kg/10 ml) continuously for two days. Colchicine (0.1 mg/5 μl) was injected from the cannula on Day 2 of DOX treatment under anesthesia. Twenty-four hours after colchicine injection, the rats were perfused with 4% PFA containing 0.2% picric acid. Then, 40-μm-thick brain sections were cut using a freezing microtome. Sections at 160 μm intervals between −1.3 and −2.0 mm from bregma were used for double staining EGFP, Oxt, AVP and CRH. The brain sections were pretreated with TrueBlack (Biotium), following which they sections incubated with blocking solution (2% bovine serum albumin, 5% normal goat serum) for 1 h. After blocking, the sections were incubated overnight at 4 °C with rabbit anti-EGFP antibody (1:1000, A-6455, Thermofisher) and either mouse anti-oxytocin monoclonal antibody (1:1000, MAB5296, Millipore), mouse anti-arginine vasopressin monoclonal antibody (1:50, ABIN109869, antibodies), or mouse anti-Corticotropin Releasing Factor monoclonal antibody (1:80, ab35748, Abcam). The sections were then rinsed with PBS and incubated with Alexa Fluor 488-labeled goat anti-rabbit IgG (1:500: Life Technologies) and Alexa Fluor 594-labeled goat anti-mouse IgG (1:500 Life Technologies) for 40 min. Next, the sections were rinsed and mounted with a mounting medium (Dako). Regarding the calculation of cell populations in the immunostained sections, PVN (160 μm intervals between −1.3 and −2.0 mm from bregma) images were obtained using a confocal laser scanning microscope (FV10i, Olympus). Therefore, four to five sections were analyzed in each mouse. The total numbers of EGFP-positive neurons, EGFP+ Oxt-positive neurons, EGFP+ AVP-positive neurons and EGFP+ CRH-positive neurons were counted in each section. The percentages of Oxt-, AVP- and CRH-positive neurons to EGFP neurons were calculated using the following equation: (the number of double-positive neurons/EGFP-positive neurons) × 100. The percentages of Oxt, AVP and CRH neurons to EGFP neurons were calculated in individual rats. Averages of the percentages obtained from individual rats were treated as data.

### C-Fos staining after intra-PVN glutamate injection

Habituated HiRet-TRE-EGFP.eTeNT- and AAV2-CMV-rtTAV16-infected rats with intra PVN cannulation (28 weeks old) were divided into three groups: first, the intra-PVN saline injection without DOX group; second, the intra-PVN glutamate injection without DOX treatment; and third, the intra-PVN glutamate injection with DOX treatment. These rats were intraperitoneally injected with doxycycline (DOX) (SIGMA-ALDRICH; 10 mg/kg/10 ml) or saline for four continuous days. At Day 4 of DOX or saline treatment, food was removed at 17:00, 24 hours before the experiment. On the experiment day, control saline or glutamate (1 μg/0.5 μl) was injected into the unilateral PVN from 10:00 to 11:45. NMDA glutamate receptors are abundantly expressed in the PVN^46^. The glutamate dose was the same as previously reported^47,48^. Two hours after injection, the rats were perfused with 4% PFA containing 0.2% picric acid. Then, 40-μm-thick brain sections were cut using a freezing microtome. Sections at 160 μm intervals between −13.3 and −14.3 mm from bregma were used for c-Fos immunostaining. The sections were rinsed in phosphate buffered saline (PBS: 0.01 M, pH 7.4) and incubated in 0.3% H_2_O_2_ for 20 min. After rinsing, the sections were incubated with PBS containing 0.3% Triton-X (T-PBS) for 30 min and incubated with 0.3% T-PBS containing 2% normal goat serum (NGS) and 2% bovine serum albumin (BSA). The sections were then incubated with rabbit anti-c-Fos antibody (RPCA-c-Fos AP; Encor Biotechnology Inc. FLA) at 1:2000 dilution for 18 h at 4 °C. After rinsing in PBS, all sections were incubated for 40 min with a biotinylated goat anti-rabbit IgG (Vector Laboratories Inc., CA) diluted 1:500. Next, the sections were incubated with an avidin-biotin complex solution (ABC kit; Vector Laboratories Inc., CA) for 1 h. Immunoreactions were visualized by incubation in nickel-diaminobenzidine (DAB; Dojin Laboratories, Kumamoto, Japan) solution (0.3% nickel ammonium sulfate, 0.02% DAB and 0.0045% H_2_O_2_ in Tris-HCl buffer [0.05 M, pH 7.4]). The number of c-Fos-positive cells per section was counted for NTS, and these numbers were averaged for all sections of the NTS in each animal and used as an individual datum.

### Measurement of BW and food intake, rectal temperature during doxycycline treatment

At 26, 35, 39, 44, 53, and 67 weeks old, doxycycline (DOX) (SIGMA-ALDRICH; 10 mg/kg/10 ml), or vehicle (saline) as the control, was i.p. injected 3 hours before the dark cycle. DOX injection was performed after BW measurement. In order to minimize the effect of stress from the i.p. injection on BW change, we determined the duration of the experimental period to be five days. We confirmed that this dose of DOX had no effect on the amount of food intake (Supplementary Fig. S9a–c). Food intake and BW were then measured during the five days of DOX injection.

Regarding measurement of rectal temperature, habituated >60-week-old rats injected with HiRet-TRE-EGFP.eTeNT and AAV2-CMV-rtTAV16 were used. Their rectal temperatures were measured before the injection of saline or DOX on Day 0 at 16:00. The rectal temperatures were then measured again on Day 3 at 16:00, and were compared with the temperatures taken on Day 0 at 16:00. Room temperature was 22.5 ± 0.5 °C. Rectal temperature measurement was performed using a thermometer (Weighing Environment Logger with rat probe: AD-1687; A&D Company Limited, Tokyo, Japan).

### Electrophysiology

Non-DOX-treated rats aged <30 weeks and >60 weeks were used in this electrophysiological analysis. Whole-cell recordings were made using an EPC 800 patch clamp amplifier (HEKA) with filtering at 1 KHz using 4–6 MΩ electrodes. Coronal brain slices (300 μm) were prepared in an ice-cold solution containing (in mM) 230 sucrose, 2 KCl, 1 KH_2_PO_4_, 0.5 CaCl_2_, 1 MgCl_2_, 26 NaHCO_3_, and 10 D-glucose. All slices were prepared at around 10:00 am to minimize the effect of circadian rhythm on the membrane potential. The slices were recovered in artificial cerebral spinal fluid (aCSF), gassed with 95% O_2_ and 5% CO_2_, containing (in mM) 126 NaCl 2.5 KCl, 1.2 MgCl_2_, 2.4 CaCl_2_, 1.2 NaH_2_PO_4_, 21.4 NaHCO_3_, and 10 D-glucose. Patch electrodes were filled with an internal solution containing (in mM) 120 K-gluconate, 10 KCl, 10 HEPES, 5 EGTA, 0.3 CaCl_2_, 1 MgCl_2_, 2 Mg-ATP, and 1 Na-GTP at pH 7.3 adjusted with KOH. The brain slices were then transferred to a recording chamber and continuously perfused at 2–4 ml/min with gassed aCSF. Whole-cell patch recordings were performed in a current clamp with a zero holding current. The membrane potentials of firing neurons were determined from slow time-scale recordings with a clear baseline. NPY (10^−8^ M: Peptide Institute Inc.) and BIBP3226 trifluoroacetate (10^−6^ M: BACHEM) were applied as shown in Figs. 3d,e,h and 4g. Data were analyzed using Clampfit software (Molecular devices).

### Immunostaining of NPY in the PVN

Rats aged 18–23 (around 20) weeks and 39–44 (around 40) weeks old were perfused with 4% PFA and 0.2% picric acid. In order to avoid effects due to circadian rhythm, all animals were perfused from 14:00 to 15:00. Brain sections of 40-μm thickness were then cut using a freezing microtome. Sections at 160 μm intervals between −1.3 and −2.0 mm from bregma were used for immunostaining. The sections were incubated with a blocking solution (PBS containing 0.3% triton, 2% BSA and 5% normal goat serum) for 60 min, then incubated with a blocking solution containing rabbit anti-NPY antibody (1:1000, Immunostar Inc.) overnight. Next, the sections were rinsed and incubated with PBS containing 2% BSA, 5% NGS, and anti-rabbit Alexa Fluor 488 (1:500, Invitrogen) for 30 min. Finally, the sections were mounted on glass slides. In order to perform the quantitative analysis, all slices were simultaneously stained under the same conditions, and all confocal images were acquired under the same fluorescence conditions (FV10i, Olympus). The brightness that reflected immunoreactions of the NPY terminals/fibers was measured by NIH image software (Image J, National Institute of Health).

### Statistical analysis

All data are presented as mean ± s.e.m. The comparison of CTB-positive neurons in the PVN projecting to the DVC in each region were analyzed by one-way ANOVA followed by Tukey’s multiple range test. Two-way ANOVA followed by Tukey’s multiple range test was used to compare BW, BW gain, and food intake each day during DOX treatment. The comparison of BW gain per amount of food intake between the control and DOX treatment groups was analyzed by using an unpaired *t*-test. The comparison of the relative brightness in the PVN between the approximately 20- and 40-week-old rats was also performed using an unpaired *t*-test. Evaluation of the effects of BIBP3226 and NPY on the PVN neurons and rectal temperature after DOX treatment was performed using a paired t-*t*est.

## Supplementary information

Supplementary Figures
