# Supplementary material for: The hypothalamus to brainstem circuit suppresses late-onset body weight gain
Source: Sci Rep. 2019 Dec 4;9:18360. doi: 10.1038/s41598-019-54870-z (PMC6892811; doi:10.1038/s41598-019-54870-z)
Supplement: Supplementary file 1 — Supplementary Figures [file 41598_2019_54870_MOESM1_ESM.pdf]

SUPPLEMENTRY INFORMATION

The hypothalamus to brainstem circuit suppresses late-onset body weight gain

Yuko Maejima, Shigeki Kato, Shoichiro Horita, Yoichi Ueta, Seiichi Takenoshita, Kazuto Kobayashi, Kenju Shimomura

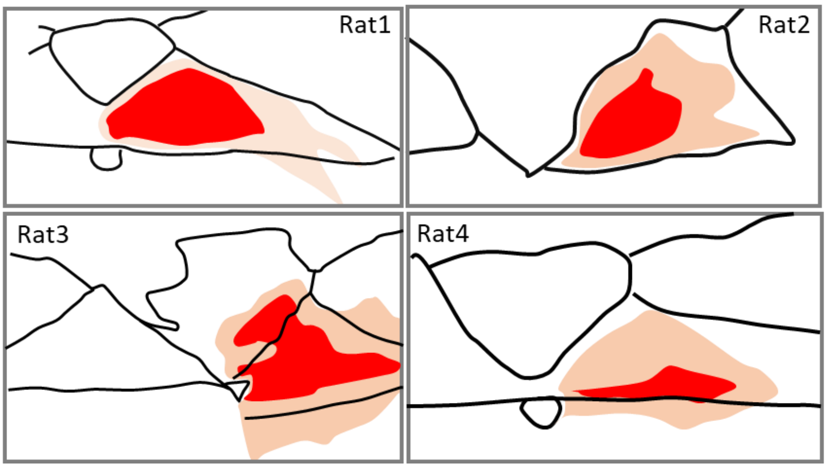

Supplementary Figure S1

CTB injection site and extent of CTB diffusion in NTS of each rat.
The red areas indicate relatively strong fluorescence intensity areas. The pale-colored areas indicate CTB diffusion. The fluorescence image used in Figure 1a corresponds to Rat4.

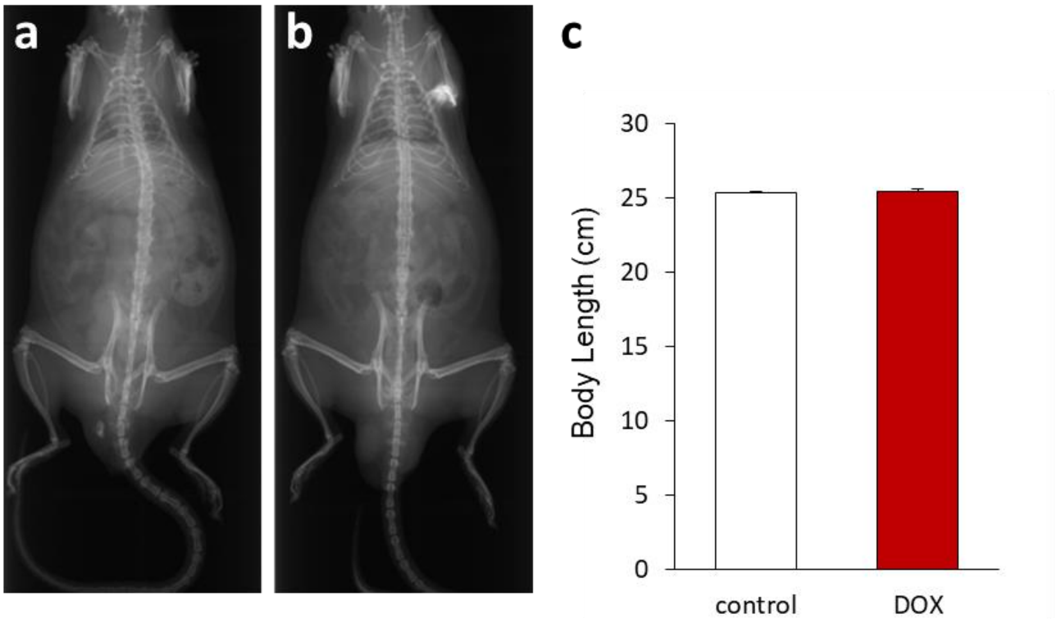

Supplementary Figure S2

Specific depression of the PVN-DVC circuit has no effect on body length.

The effect of blocking the PVN-DVC circuit on body length was examined using X-ray skeleton images. Rats aged 87 weeks were anesthetized by isoflurane after multiple DOX injections (main Fig. 1l) and their body lengths (from the tip of the nose to the anus) were measured. X-ray images were also acquired (La Theta LCT-200; Hitachi Aloka Medical).**a–b**: Representative X-ray images of a rats after saline injection (control) (a) and multiple DOX injections (b). No obvious differences were detected in the X-ray images. **c:** Comparison of body length between the control group (25.34 ± 0.09 cm) and the DOX group (25.47 ± 0.11 cm). There were no significant differences in body length. Unpaired *t*-test. $n = 5, 6$.

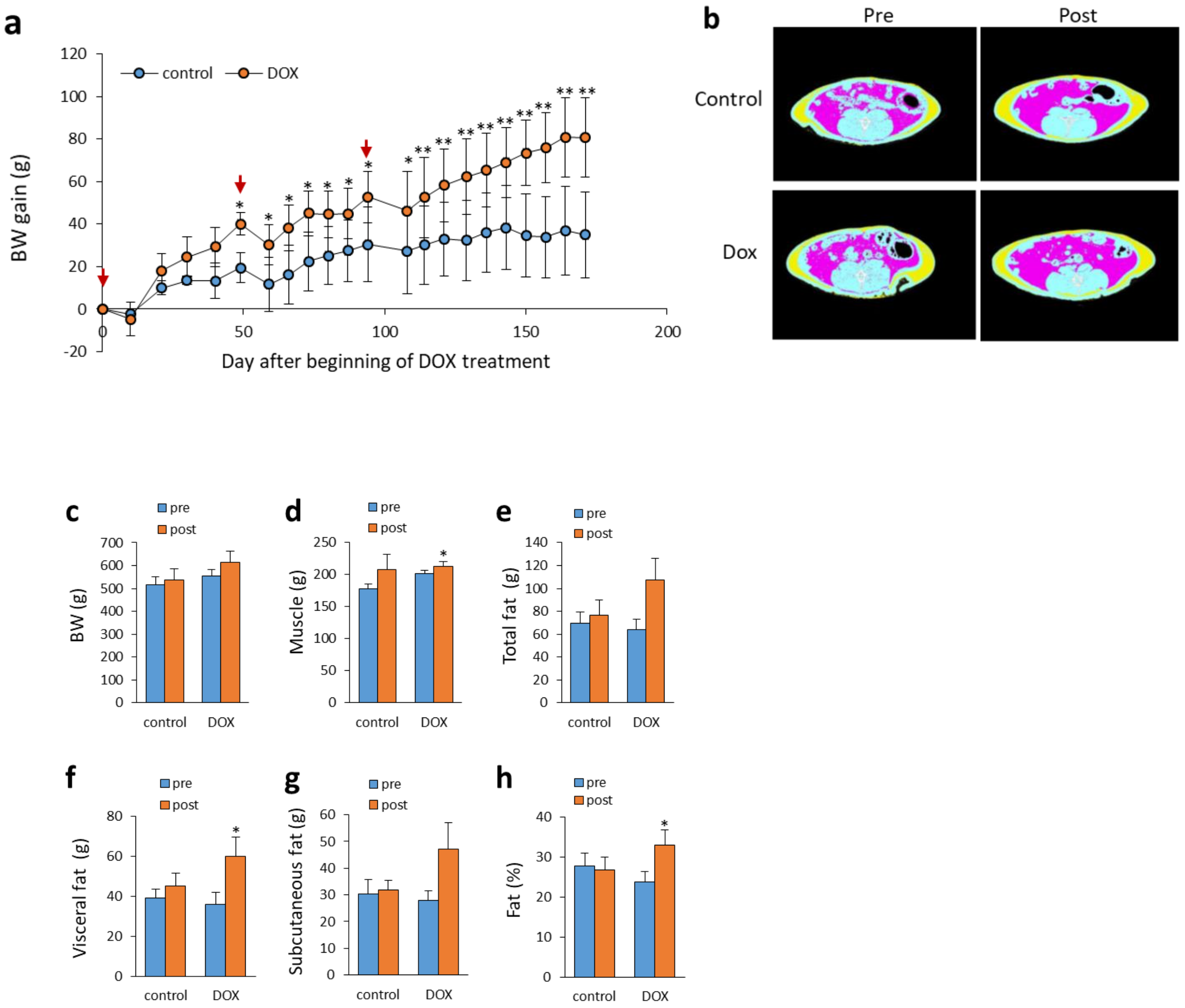

Supplementary Figure S3

Comparison of body composition before and after DOX treatment.

The effect of blocking the PVN-DVC circuit on body weight gain and levels for fat and muscle were examined in the aged rats. The rats aged 47–59 weeks old were injected with HiRet-TRE-EGFP.eTeNT, into the NTS. After two weeks, these rats were then injected with AAV2-CMV-rtTAV16, into the PVN. The first DOX (at Day 0) was injected at age 55–67 weeks. **a,** BW changes after treatment of saline (control) or DOX. The red arrows in the figure indicate the first of five consecuative DOX treatments ($F_{1, 84}$ = 156.41, P < 0.01) Error bars indicate s.e.m * P < 0.05, ** P < 0.01, Two-way ANOVA followed by Tukey's multiple range test. (*n* = 3, 3). **b,** Representative CT scanning images before and after saline or DOX injections. Yellow and pink colored area indicate subcutaneous fat and visceral fat, respectively. CT images before treatment were captured at age 55–67 weeks. CT images after treatment were captured at age 83–95 weeks. **c–h**, The comparison of BW (c), muscle (d), total fat (e), visceral fat (f), subcutaneous fat (g) and fat percentage (h) between before and after DOX treatment in the control and DOX-treated groups. * P < 0.05. Paired t-test. (*n* = 3, 3). Error bars indicate s.e.m

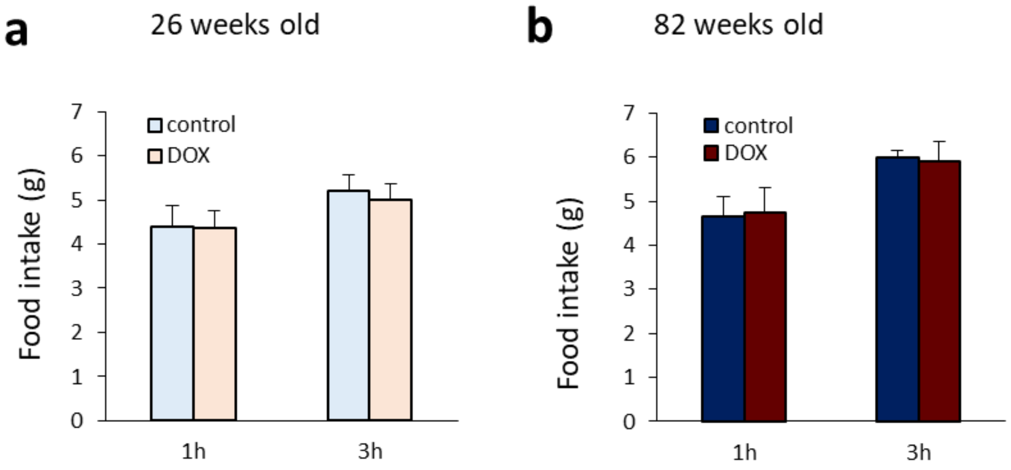

Supplementary Figure S4
Short-term food intake after fasting and re-feeding under DOX treatment in young (26-week-old) and aged (82-week-old) rats.
To examine the effects of blocking the PVN-DVC circuit on food intake on an hourly basis, food intake was measured in rats aged 26 (*n* =9, 9) and 82 (*n* =5, 6) weeks old while under DOX treatment. At Day 2 of DOX treatment, food was removed for 14 h before re-feeding, and food intake was measured at time points of 1 h and 3 h. Both the young **(a)** and adult rats **(b)** showed no significant differences in food intake at 1 h and 3 h between the control and the DOX-treated groups (**a**: ($F_{1, 16}$ = 0.14, P > 0.05), **b**: ($F_{1, 9}$ = 0.002, P > 0.05). Error bars indicate s.e.m. Statistical analyses were performed using two-way ANOVA with post hoc Tukey's test.

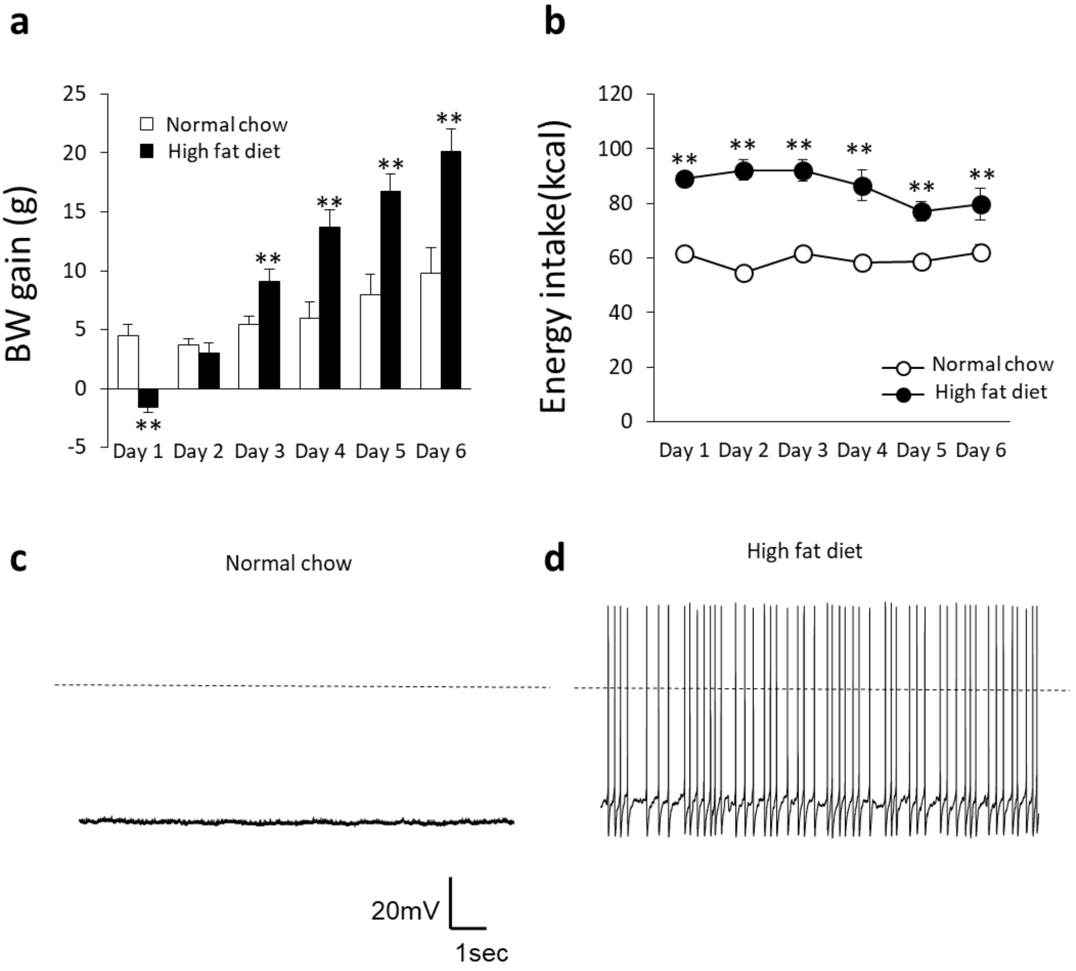

Supplementary Figure S5

The effect of high fat diet on the BW, PVN neuronal activity in young rats.

In order to examine the effect of high fat diet on the BW and neuronal activity, 14-week-old Wistar rats were fed high fat diet for six days. **a–b,** BW change (a) and energy intake (b) under high fat diet feeding. On the first day of the high fat diet, energy intake was significantly increased ($F_{1, 50} = 86.1$, $P < 0.01$), compared with the normal chow diet. However, BW change was significantly decreased ($F_{1, 50} = 308.6$, $P < 0.01$), compared with normal chow fed control. Error bars indicate s.e.m. Statistical analyses were performed using two-way ANOVA with post hoc Tukey's test.

c–d, Representative recording of neuronal activity in Oxt neurons of 14-week-old Oxt-mRFP rats under a normal chow diet (c) and high fat diet (d). Twenty-four hours after starting high fat diet feeding, the PVN Oxt neurons were remarkably activated (d). However, the PVN Oxt neurons were silent in the rat with the normal chow diet.

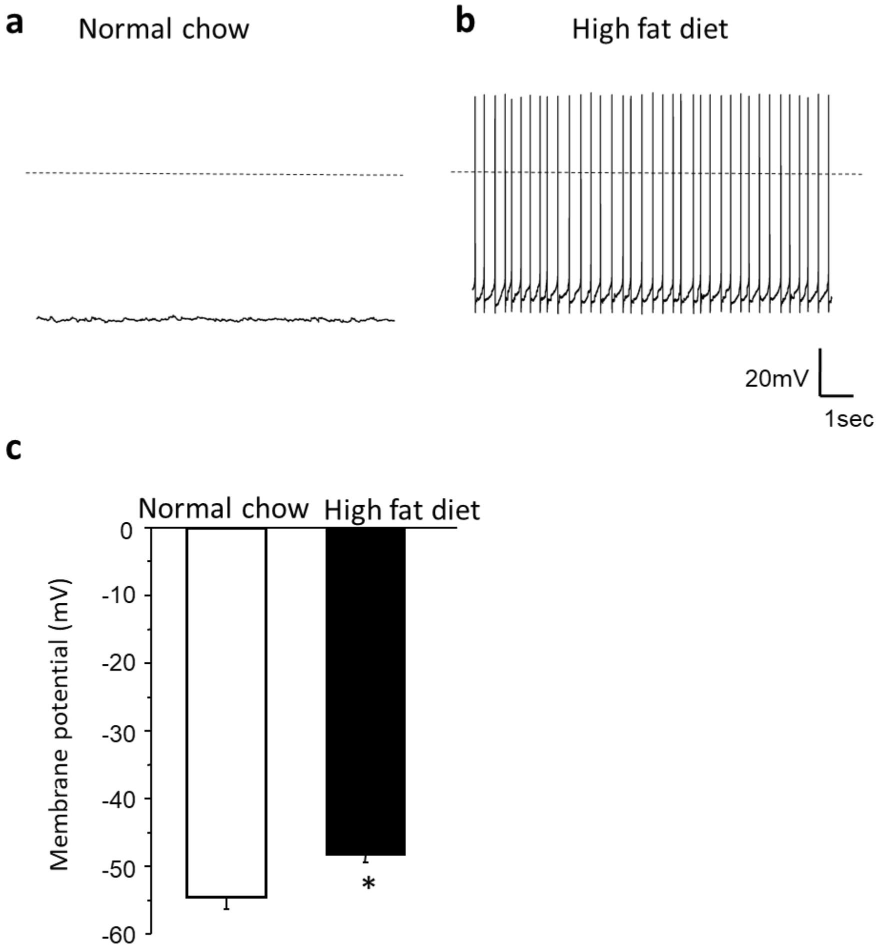

Supplementary Figure S6
The electrical activity in the PVN from young rats fed with high-fat diet.
Based on the concept that the effect of the PVN in the hypothalamus on BW regulation is to regulate normal BW, the electrical activity of PVN neurons from Oxt-mRFP rats (16 weeks old) fed with a high-fat diet (HFD) for eight weeks were examined. HFD (HFD32) and normal chow (CE2) were purchased from CLEA (Osaka. Japan) **a–b**, Representative recording of electrical activity of PVN Oxt neurons from normal chow-fed Oxt-mRFP rats (*n* = 9) (a) and HFD-fed Oxt-mRFP rats (*n* = 21) (b). The PVN Oxt neurons from the normal chow-fed Oxt-mRFP rats were inactive. However, the PVN Oxt neurons from the HFD-fed Oxt-mRFP rats were more depolarized with increased action potential firing. **c,** Membrane potential in PVN Oxt neurons from the normal chow-fed and HFD-fed rats. The membrane potential of the PVN Oxt neurons from the HFD-fed Oxt-mRFP rats was significantly higher than the normal-chow fed Oxt-mRFP diet. * $P < 0.05$. Unpaired t-test. Error bars indicate s.e.m.

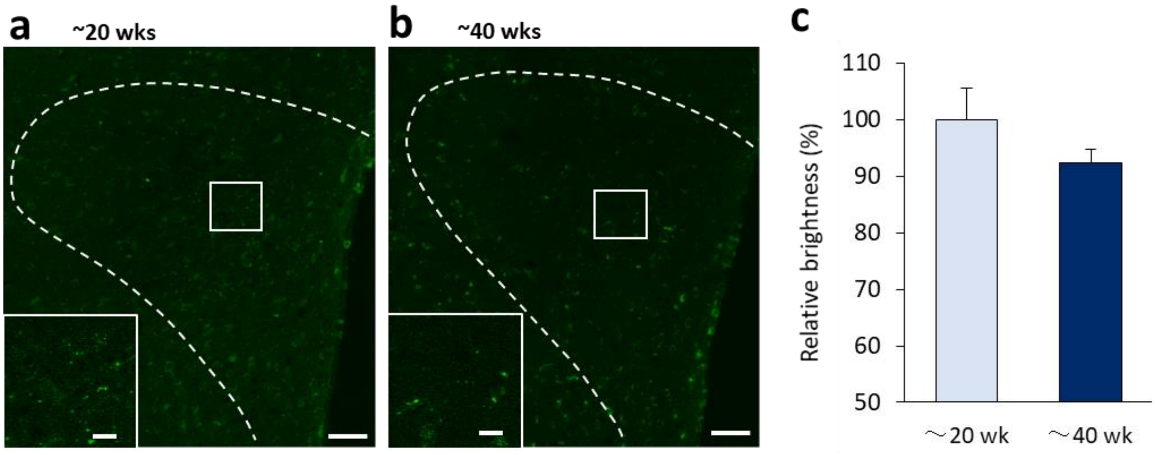

Supplementary Figure S7

Comparison of α-MSH fibers/terminals in the PVN between around 20- and 40-week-old rats.
Immunostaining of α-MSH fibers/terminals in PVN was performed in the same manner as that of the NPY fibers/terminals (see immunostaining of NPY in PVN in the main text). The primary and secondary antibodies used in this experiment were rabbit anti-α-MSH antibody (1:1000, Immunostar Inc.) and anti-rabbit Alexa Fluor 488 (1:500, Invitrogen).

a, b, Representative confocal images of α-MSH fibers/terminals in the PVNs of rats aged around 20 (a) and around 40 (b) weeks. Scale bars indicate 50 μm. The squares located in the bottom left show an enlarged image of the white squares in images (a) and (b). Scale bars located in the enlarged images indicate 10 μm. (c) Relative brightness acquired from image analysis of confocal images by using NIH image software (Image J, National Institute of Health). The brightness of α-MSH immunofluorescence in the PVN of the approximately 20-week-old rats was calculated as 100% (n = 3 each). The relative brightness in the approximately 40-week-old rats tended to decrease, but there were no significant differences. Statistical analyses were performed using an unpaired t-test.

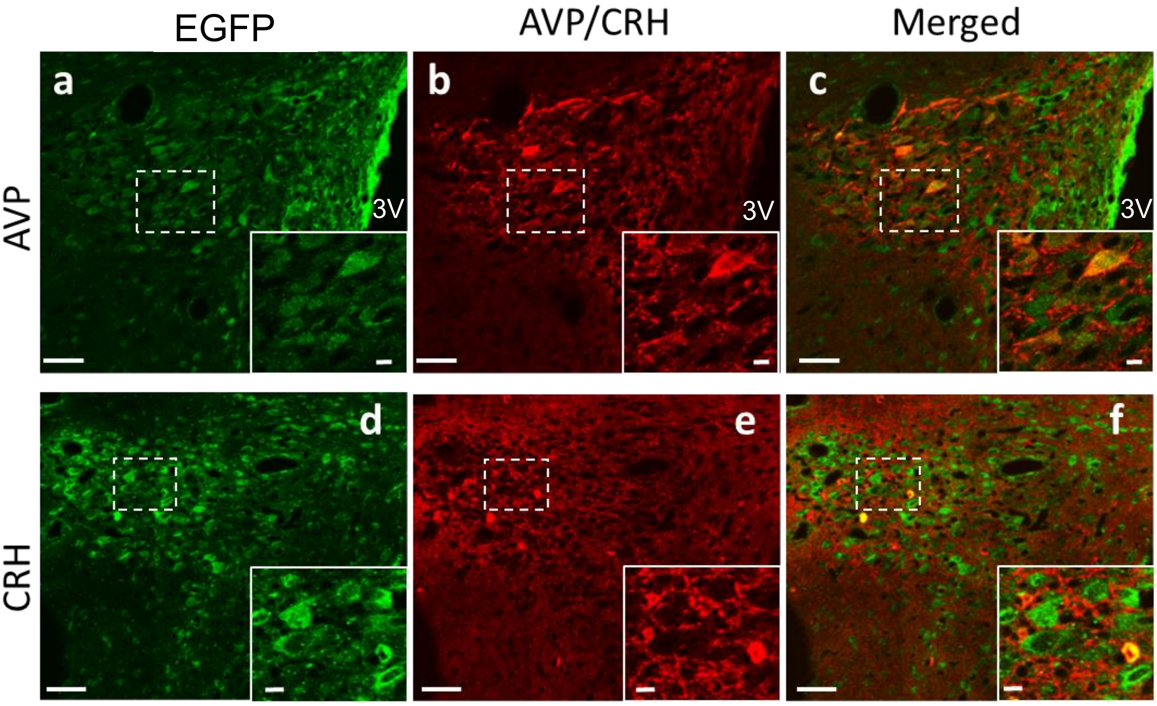

Supplementary Figure S8

AVP and CRH neurons are also one of the components of the PVN-DVC circuit.

a–c, The distribution of EGFP-expressing neurons (a), AVP-positive neurons (b) and a merged image of a and b (c) in the PVN after DOX treatment continuously for two days. Scale bars indicate 50 µm. The image located in the right bottom is an enlarged image of the dotted square in each image. Scale bars indicate 10 µm. 3V = 3rd ventricle. **d–f,** The distribution of EGFP-expressing neurons (d), CRH positive neurons (e) and merged image of d and e (f) in the PVN after DOX treatment continuously for two days. Scale bars indicate 50 µm. The image located in the right bottom is an enlarged image of the dotted square in each image. Scale bars indicate 10 µm. 3V = 3rd ventricle.

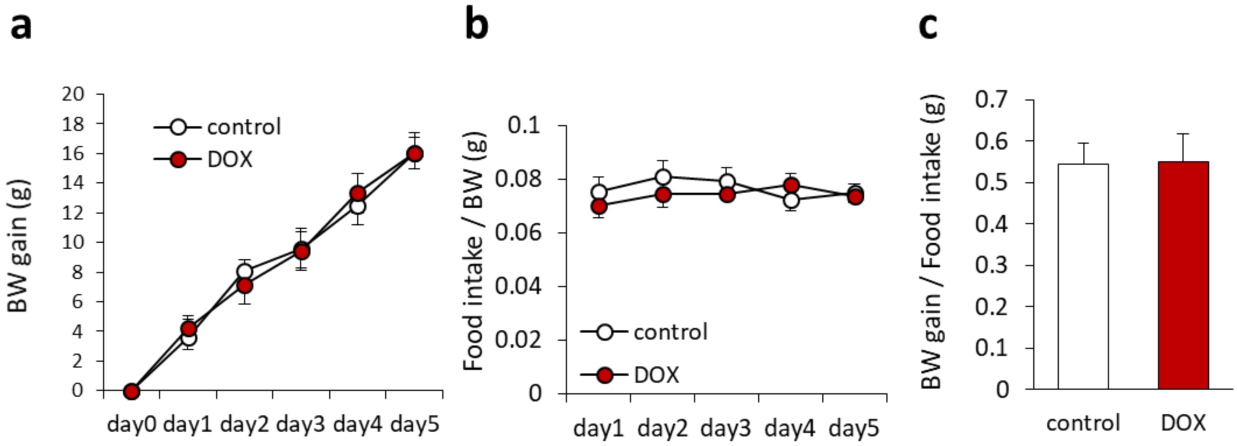

Supplementary Figure S9

The effects of doxycycline on BW gain, food intake, and food efficacy.

To examine whether DOX has direct effect on food intake or BW gain, DOX was intraperitoneally injected (SIGMA-ALDRICH: 10 mg / kg / 10 ml) into normal Wistar rats 3 h before the dark cycle for five consecuative days. **a–b,** Since the rats (8–9 weeks old) were growing, BW gain increased from Day 0 in both groups. However, there were no significant differences in BW gain and food intake between the control and DOX groups (BW gain: ($F_{1, 85} = 0.032$, $P > 0.05$, Food intake: ($F_{1, 68} = 2.525$, $P > 0.05$). Error bars indicate s.e.m. Statistical analyses were performed using a two-way ANOVA with post hoc Tukey's test. **c,** Food efficacy (BW gain / food intake) was also unaffected by DOX treatment. These data show that the dose of DOX used in this experiment had no influence on food intake or BW gain. Therefore, the data shown in this paper should be the result of specifically blocking the PVN-DVC circuit. Error bars indicate s.e.m. unpaired *t*-test. $n = 10, 9$.
